# Lectures on the Feeding of Infants

**Published:** 1904-12-24

**Authors:** James Burnet

**Affiliations:** Registrar Royal Hospital for Sick Children; Senior Clinical Tutor, Extramural Wards Royal Infirmary; Physician to The Marshall Street Dispensary, Edinburgh


### Hospital Clinics.

### LECTURES ON THE FEEDING OF INFANTS.

By James Burnet, M.A., M.B, M.R.C.P., Edin. Registrar Royal Hospital for Sick Children; Senior Clinical Tutor, Extramural Wards Royal Infirmary; Physician to The Marshall Street Dispensary, Edinburgh.

### I.—Breast Feeding and Weaning.

*Breast Feeding and Its Management.*—Every mother should nurse her child. This is a law to which no exception should be made save on the ground of ill-health. The excuse that the mother has not enough milk is very often found, on careful investigation, to be untenable. In a large number of cases when the complaint of having insufficient milk is made we find that by carefully attending to the diet and general health of the parent a good milk supply becomes established within a very short time. The diet of a nursing woman should consist partly of proteid and partly of carbohydrate. A fair amount of fluid is also necessary. It is a mistake, however, to suppose that by altering the diet we can greatly influence the milk supply. By increasing the amount of proteid taken we may raise the percentage of fat in the milk, but only to a nominal extent. If, on the other hand, too much fat be consumed we diminish the percentage of fat in the milk. The amount of proteid is best increased by allowing more nitrogenous food, and is most quickly diminished by increasing the amount of exercise taken by the patient. Malt and oatmeal gruels have a slight influence in increasing the milk supply, but they are not nearly so potent in this respect as some would lead us to believe. A useful drink for nursing mothers is made by mixing two table-spoonfuls of Mellin's food in a glass of warm milk, and this may be taken two or three times a day.

Alcohol should never be given to women who are nursing. There is not only a risk lest the child be affected thereby, but also the giving of alcohol in any form is apt to impair the quality of the milk, apart altogether from the fact that the woman may form alcoholic habits.

The breasts should be thoroughly bathed after each nursing, and the nipples smeared over with almond oil. If the nipples are painful a nipple shield is of great service, and when not in use this should be kept in a bowl containing a solution of bicarbonate of soda.

The contra-indications to breast-feeding belong to two distinct groups. There is first the group of constitutional causes. These include tuberculosis, hysteria, and the presence of any acute disease. Next comes a large group of local conditions, such as fissured nipples, mammary abscess, and cancer. Then we must remember that no child should be kept on the breast who steadily loses weight, in spite of the fact that he is being nursed regularly and carefully.

The composition of human milk has been variously stated by different authorities. It should be remembered that the composition of the milk at the beginning of the nursing act differs very considerably from its composition during its progress and at the end. The following table illustrates this point very clearly:—

| Ingredient. | At Beginning. | During Progress. | At End. |
|---|---|---|---|
| Fat ... ... | 1 70 | 2·77 | 4 51 |
| Sugar ... ... | 5 56 | 5 70 | 5·10 |
| Proteid ... ... | 1 13 | 0 94 | 0·71 |
| Ash ... ... | 0 46 | 0 32 | 0 28 |
| Water ... ... | 90 24 | 89 68 | 87 50 |

It will be at once evident that the fat is the ingredient which shows the most variableness. It is greatly increased in amount as suckling goes on. At the same time the proteids are materially diminished. The other constituents are altered but little. It is only fair to state, however, that somewhat different results have been obtained by various observers, but all are agreed that the fat is considerably increased at the end of the nursing act.

No analysis of human milk can be taken as a standard, for we find that one child thrives on milk which shows a very different percentage composition from that by which another child is nourished. It would seem as if each individual mother's milk was specially prepared to suit the wants of her own particular offspring. Hence we maintain that any artificial food which attempts to imitate in percentage composition that of average breast milk cannot possibly be expected to suit all and sundry alike. And so in practice we find that while some particular food may be taken with impunity (we do not say with advantage) by one infant, when given to another it produces evidences of malnutrition, not to speak of gastro-intestinal derangement. It is one of those fallacies which is constantly being thrust upon the profession that the food which most nearly approaches in percentage composition that of mother's milk is the ideal substitute for human milk. Could any statement be more misleading? It is quite contrary to facts, and in practice proves most disastrous and injurious.

Provided the mother can be got to nurse her child, certain points require attention if breast-feeding is to prove successful. First of all, there must be regularity in nursing. Infants are automatic in their movements, and they can be made quite as automatic in respect to their desire for food. The child must neither be fed too often, nor yet irregularly. Too frequent feeding leads to the milk becoming highly indigestible, owing to great increase in its proteids. Roughly speaking, the child should be fed every two hours for the first month, every two and a half hours during the second and third, and every three hours after this until he is nine months old, when, as a rule, he may be weaned.

The mother will often ask, "Should the baby be waked up to be fed?" The reply should always be,

"Yes." If this is done at the start the child will soon learn to wake up at regular intervals of itself. As to the length of time allowed for each nursing 15 minutes is long enough, and the child should never be allowed to fall asleep at the breast. After nursing he should never be rocked or shaken about, as these unnecessary movements tend to cause regurgitation from the stomach. He should, therefore, be placed quietly in his crib and allowed to sleep until the next nursing period comes round. If fed at 10.30 or 11 P.M. the infant should sleep till 4.30 or 5 A.M., although in the case of weakly children it may be necessary to give the breast once between these hours. On the other hand, many healthy babies sleep from 10.30 P.M. till seven in the morning without being fed.

While nursing, the mother should take a certain amount of outdoor exercise every day. She should retire to rest not later than 10.30, and should not engage in dancing or any violent form of exercise. She should also guard carefully against constipation, as this condition is apt to lead to a similar state in the nursling, and indeed may induce a permanently more or less chronic condition which is troublesome as the child grows older.

The process of weaning is always a somewhat troublesome one. Generally speaking the ninth month is the most suitable time for beginning the weaning process which should always, if possible, be brought about gradually. If, however, the child is suffering from any acute illness the date of weaning should be put off for a time. It is also advisable to avoid weaning during the late summer and early autumn months as cow's milk may lead to vomiting and diarrhœa at these times.

The process of weaning should occupy at least a month. At first one feeding should be given, preferably with a spoon. This may be given in the forenoon. Later on a second feeding may be added in the evening. Later still the breast-feeding should only be allowed three or four times in the day, and then gradually reduced until the child is entirely being fed on cow's milk. By following this plan the stomach and intestines gradually accommodate themselves to the digestion of this mixed form of feeding. A suitable milk mixture to begin with is one which is somewhat dilute, and we generally use the following as a start :—Milk, six tablespoonfuls ; barley-water, seven and a half tablespoonfuls ; cream, one large teaspoonful ; ordinary sugar, one small teaspoonful. It is a mistake to make the mixture too strong, and we often find it advantageous to still further dilute it by giving five and a half tablespoonfuls of milk, and eight of barley-water.

Difficulties, of course, may arise in attempting to wean the child. Very commonly he will refuse everything but the breast. The only remedy for this is to withhold the breast, if necessary, for 24 hours, and insist on the child taking cow's milk. Sometimes spoon-feeding is difficult, and a bottle must be substituted. This is quite allowable if a bottle with a plain rubber teat is employed. Two bottles will be required, one for immediate use, and the other to be kept in soda solution. Gastro-intestinal disturbances are common at first, but these can usually be got over by altering the composition of the mixture. It is often the proteid that is at fault, and by diluting the milk this difficulty is usually overcome. Sometimes small doses of calomel and soda prove beneficial, but medication should not be resorted to in the first instance.

When the mother is unable to nurse her child a wet nurse is sometimes employed. This practice, however, is now dying out ; and we need not, therefore, refer to it at any great length. The value of wet-nursing is extremely discounted if we remember, what has been already clearly pointed out, that no two women's milks are alike in composition, and that every mother's milk is so constituted as to suit the needs of her offspring and hers alone. Apart from this wet nurses can usually only be drawn from a class of women whose presence in a private house is most undesirable.

*Weight and Growth in Relation to Breast-Feeding.* Much difference of opinion exists as to what may be considered the normal average weight at any particular month during the period of infancy. We know that the weight is always greater at night than in the morning, and therefore the infant's weight should always be recorded at exactly the same hour each day. So far as we can learn the average gain in weight during the first year of life should be as follows :—

| | Gain per week. |
|---|---|
| During the first three months ... | ... 6 ounces |
| During the second three months | ... 5 ounces |
| During the third three months ... | ... 3 ounces |
| During the fourth three months | ... 2 ounces |

A loss in weight should always attract the physician's attention, as no child who is steadily gaining weight can be anything but healthy. Even a failure to gain weight during any particular period is suspicious, though probably not of so much immediate importance as an actual loss of weight.

Whenever we find a child ceasing to gain in weight or losing we should inquire first as to the mother's state of health and as to the condition of her milk and her habits in nursing. If these are found to be satisfactory then we must turn our attention to the infant. Very often serious illness may be averted by placing the child immediately under proper treatment as soon as its weight is observed to be unsatisfactory.

Growth is also of some importance. Stunted growth means some nutritional disturbance and in not a few cases is associated with mental conditions. This is especially true of those infants who appear healthy enough although they do not seem to lose their babyish stature. Thus cretins and the subjects of primary or secondary amentia are usually small and undeveloped, so also are microcephalic idiots. In general, therefore, we should pay attention to the child's development, as thereby we may obtain information which will guide us in the future management and treatment of the case.

